# Comprehensive Analysis of Rice Laccase Gene (*OsLAC*) Family and Ectopic Expression of *OsLAC10* Enhances Tolerance to Copper Stress in *Arabidopsis*

**DOI:** 10.3390/ijms18020209

**Published:** 2017-01-30

**Authors:** Qingquan Liu, Le Luo, Xiaoxiao Wang, Zhenguo Shen, Luqing Zheng

**Affiliations:** 1 College of Life Sciences, Nanjing Agricultural University, Nanjing 210095, China; liuqingquan@cnbg.net (Q.L.); 2014116043@njau.edu.cn (X.W.); zgshen@njau.edu.cn (Z.S.); 2 Institute of Botany, Jiangsu Province and Chinese Academy of Sciences, Nanjing 210014, China; 3 College of Resources and Environmental Sciences, Nanjing Agricultural University, Nanjing 210095, China; luole@njau.edu.cn

**Keywords:** rice laccase, *OsLAC10*, copper tolerance, copper uptake, *Arabidopsis*

## Abstract

Laccases are encoded by a multigene family and widely distributed in plant genomes where they play roles oxidizing monolignols to produce higher-order lignin involved in plant development and stress responses. We identified 30 laccase genes (*OsLACs*) from rice, which can be divided into five subfamilies, mostly expressed during early development of the endosperm, growing roots, and stems. *OsLACs* can be induced by hormones, salt, drought, and heavy metals stresses. The expression level of *OsLAC10* increased 1200-fold after treatment with 20 μM Cu for 12 h. The laccase activities of OsLAC10 were confirmed in an *Escherichia coli* expression system. Lignin accumulation increased in the roots of *Arabidopsis* over-expressing *OsLAC10* (*OsLAC10*-OX) compared to wild-type controls. After growth on 1/2 Murashige and Skoog (MS) medium containing toxic levels of Cu for seven days, roots of the *OsLAC10*-OX lines were significantly longer than those of the wild type. Compared to control plants, the Cu concentration decreased significantly in roots of the *OsLAC10*-OX line under hydroponic conditions. These results provided insights into the evolutionary expansion and functional divergence of *OsLAC* family. In addition, *OsLAC10* is likely involved in lignin biosynthesis, and reduces the uptake of Cu into roots required for *Arabidopsis* to develop tolerance to Cu.

## 1. Introduction

Laccase (EC 1.10.3.2) was originally found in *Rhus vernicifera* by Yoshida [1] and belongs to the ceruloplasmin oxidase family. Laccase has three catalytic sites that combine with four copper (Cu) ions, and catalytically oxidize various aromatic and non-aromatic compounds in the presence of oxygen. Some compounds such as Cu chelators, azides and fatty acids, and hexadecyl trimethyl ammonium bromide (CTAB) inhibit laccase activity by altering the spatial structure of the enzyme or the binding site for the substrate [2].

Laccase is widely present in plants and fungi but is also found in bacteria and insects [3,4]. Many studies have been conducted on fungal laccase, whereas studies on laccases in higher plants are limited and have considered disease resistance and lignin biosynthesis. Laccase has been isolated and identified in *Arabidopsis thaliana*, rice, tobacco, ryegrass, cotton, boxwood, poplar, and sycamore maple [5,6,7,8,9,10,11]. The most detailed study of laccase was on the *Rhus vernicifera* laccase [12].

Numerous in vitro tests have shown that laccases and peroxidases are involved in the polymerization of monomers [5,13], but research on the role of laccase in lignin biosynthesis in plants remains limited. Plant laccase genes belong to a large, widespread family in higher plants and some lower plants, such as mosses and algae. Some laccase genes in the dicot model plant *Arabidopsis* are involved in lignin biosynthesis. One study showed that 8 of 17 *Arabidopsis* laccase genes are highly expressed in the inflorescence stems, which may participate in lignin polymerization, and found that lignin content produced by two laccase genes (*lac4* and *lac17*) in a double-knockout *Arabidopsis* mutant was less than that of wild-type, demonstrating that laccases are involved in oxidizing lignin polymers in plants [14]. Subsequently, Zhao et al. found that lignin content in *Arabidopsis lac11 lac4 lac17* triple-mutant roots is almost undetectable, indicating that *AtLAC11* lignin may also be involved in lignin polymerization [15].

On the other hand, biotic and abiotic stresses can change the lignin composition of plants. Some laccase genes are expressed in non-woody tissues, and laccase also participates in the oxidation of flavonoids, suggesting that laccase plays an important role in plant growth and defense responses [16,17]. However, the roles of lignin biosynthesis-related laccase genes in response to environmental stressors have not been reported. Only *AtLAC2* has been shown to be involved in *Arabidopsis* responses to drought stress [18], as other laccase genes have rarely been reported to be involved in the responses to environmental stress [19,20]. A more comprehensive understanding of this family of laccase genes is needed to better understand the function of laccase.

Research on laccase has mainly focused on the function of enzymes encoding lignin biosynthetic genes and the mechanisms of laccase gene expression in response to environmental stress [14,15,18,19,21]. Few detailed studies on the genetics of biological functions have been performed. An important role of lignin biosynthesis in the response of plants to environmental stress has been reported and laccase may participate in the response to environmental stress by affecting lignin accumulation or its oxidative functions [22].

Copper (Cu) is one of the essential microelements for plant growth and development, playing important roles in photosynthesis, respiration, and C and N metabolism. Cu is toxic to plants when in excess. Cu inhibits root growth and causes oxidative damage [23]. Excess Cu can be detoxified through complexation with organic ligands, such as organic acids, amino acid, and metal-binding peptides. Cu can also be bound with cell walls, which prevents its entry into the cytosol [24].

In general, solutes such as metals have to be taken up into the exodermis and go through the cortex and endodermis before they enter the xylem in plant roots [25]. Through long-distance transport, Cu will be send to its function organ, tissue or cells. The transport of Cu is generally a tightly controlled process mediated by multiple Cu transporters, such as Copper transporter (COPT), heavy metal ATPase (HMA), Zrt/Irt-like protein (ZIP), natural resistance-associated macrophage protein (Nramp) and yellow stripe-like (YSL) transporters, which were involved in maintaining Cu homeostasis [26,27,28,29,30]. Xylem-unloading processes are very important step in controlled distribution and detoxification of metals in the shoot [31]. However, the root-to-shoot translocation of Cu is limited, probably because of a strong accumulation in the cell walls of the cortex, where its concentration decreases from the outer to the inner cell layers [32].

In a previous study, we found that Cu stress significantly enhanced laccase activity and lignin deposition in rice roots [33], and we speculated that laccase plays an important role in plant tolerance to Cu. In-depth molecular-level research is needed to better understand the function of rice laccase. In this study, we analyzed the basic characteristics of rice laccase genes and their expression patterns using the bioinformatics method, and found a Cu-induced rice laccase gene (*OsLAC10*) by RNA-Seq and quantitative reverse transcription-polymerase chain reaction (qRT-PCR) analyses. Ectopic expression of *OsLAC10* in *Arabidopsis* resulted in enhanced tolerance to Cu stress.

## 2. Results

### 2.1. General Information on the Rice Laccase Gene Family

**Figure 1:**
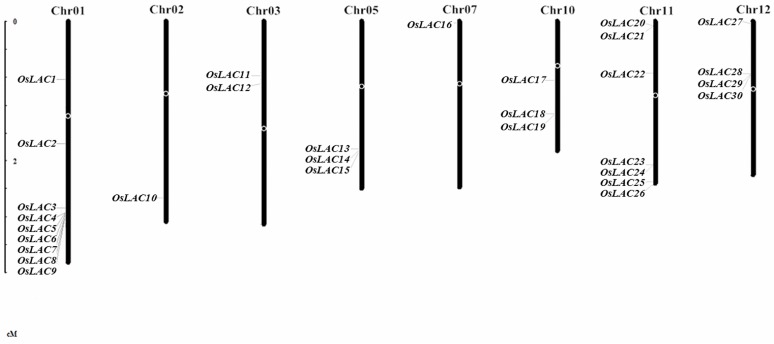
Chromosome map depicting location of rice laccase genes.

We BLAST (Basic Local Alignment Search Tool) searched the National Center of Biotechnology Information (NCBI), Universal Protein (Uniprot), Rice Genome Annotation Project (RGAP), and the Rice Annotation Project Database (RAP-DB) for the conserved amino acid sequence of *Arabidopsis* laccase, and found 30 laccase genes in the rice genome, which are located on eight chromosomes (Figure 1). The subcellular prediction showed that most of the rice laccase proteins are localized in the secretory pathway and a few are located in mitochondria or chloroplasts, indicating that most rice laccases are extracellular proteins. A signal peptide analysis showed that most of the laccases are secretory proteins, but some, such as OsLAC13 and OsLAC21, have no signal peptide or glycosylation sites, indicating that they may be intracellularly localized. Like most *Arabidopsis* laccases, most secretory rice laccases are *N*-glycosylated glycoproteins (Table 1).

### 2.2. Phylogenetic Analysis of the Rice Laccase Family

**Figure 2:**
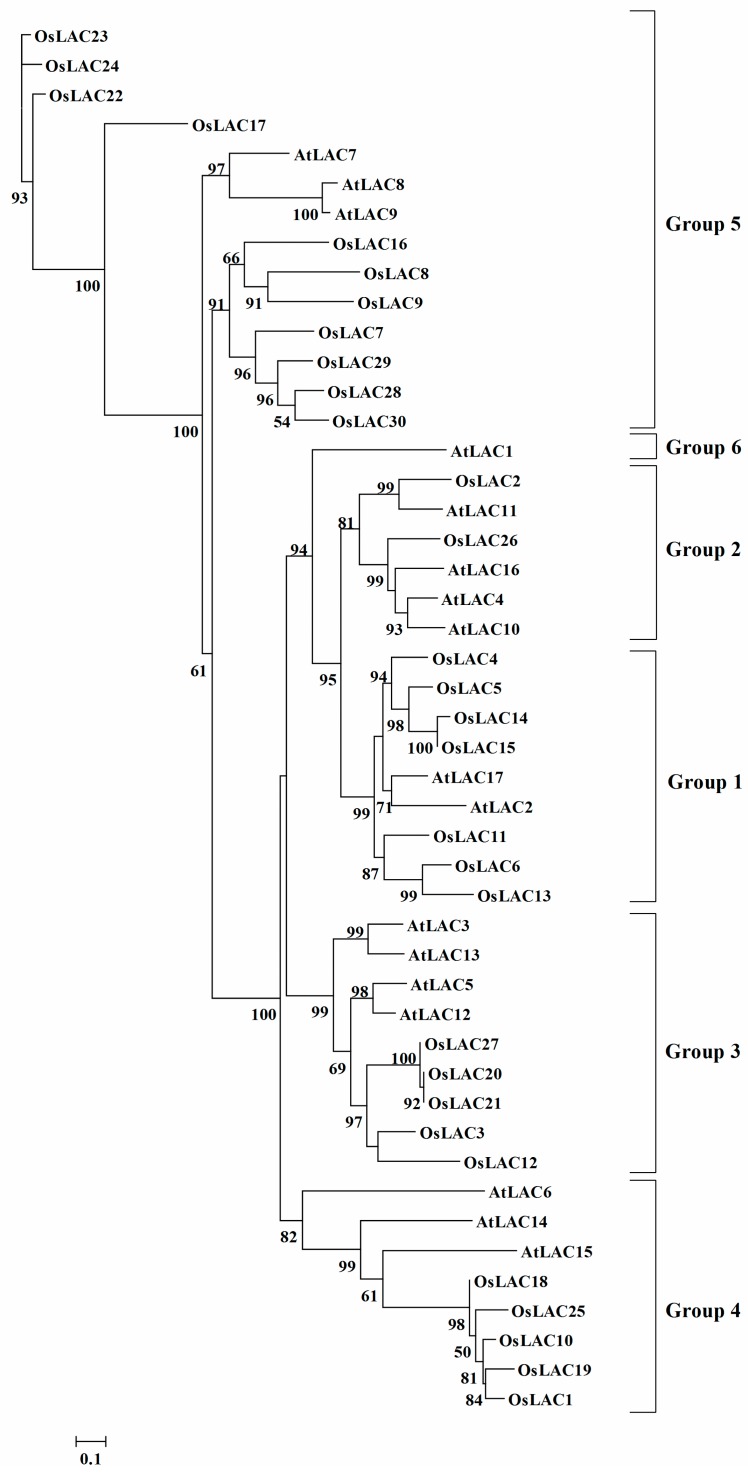
Phylogenetic tree of the rice and *Arabidopsis* laccases obtained by protein sequence alignment of rice and *Arabidopsis* laccases.

The sequence identity among individual members of rice laccase family is low (Table S1). An unrooted phylogenetic tree of rice and *Arabidopsis* laccases was generated from their respective amino acid sequence alignments (Figure 2). According to the classification standard of *Arabidopsis* laccases [11], 30 rice laccase members were divided into five groups, with seven members in group 1, two in group 2, five in group 3, five in group 4, and 11 in group 5. Group 6 contained only one *Arabidopsis* laccase (AtLAC1). Although the Cu ion binding domain of rice laccase was more conserved in the phylogenetic analysis, kinship among these laccase members was distant, indicating a diversity of functions.

### 2.3. Spatiotemporal and Abiotic Stress-Inducible Expression Patterns of Rice Laccase Genes

**Figure 3:**
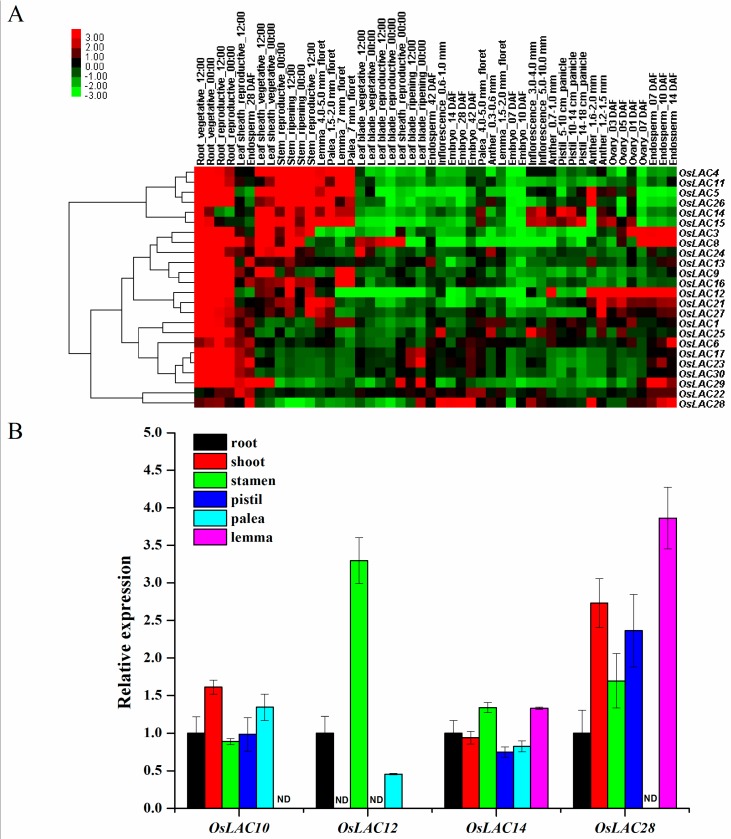
Expression patterns of rice laccase (*LAC*) genes in various tissues/organs and developmental stages. (**A**) Cluster heat map for spatiotemporal location of laccase genes expressions in various tissues/organs of rice. Normalized signal intensity of genes obtained from the RiceXPro database; (**B**) Real-time PCR analysis of selected genes to validate their differential expression during various stages of development. The mRNA levels for each gene in different tissue samples were calculated relative to its expression in root. The error bars represent standard deviation. ND, not detected.

To understand the expression patterns of rice laccase genes in different tissues and at different growth stages, we determined their spatiotemporal expressions based on the RiceXPro database. A clustering heat map showed that most rice laccase genes were highly expressed in the roots (including vegetative and reproductive growth stages). Others, mainly from the group 1 subfamily, were also highly expressed in the stems. Some laccase genes were highly expressed in the endosperm, including *OsLAC3*, *OsLAC8*, *OsLAC12*, *OsLAC28*, and *OsLAC29.* Two genes, *OsLAC14* and *OsLAC15* were highly expressed in the flowers (pistil, palea and lemma) (Figure 3A). The differential expression of representative *LAC* genes was also confirmed by qRT-PCR analysis (Figure 3B). Although the expression levels of some genes obtained by microarray or qRT-PCR were slightly different, the variation tendencies of the examined genes were generally similar. In summary, rice laccase genes were mainly expressed in the endosperm, vegetative roots, stems and flowers.

**Figure 4:**
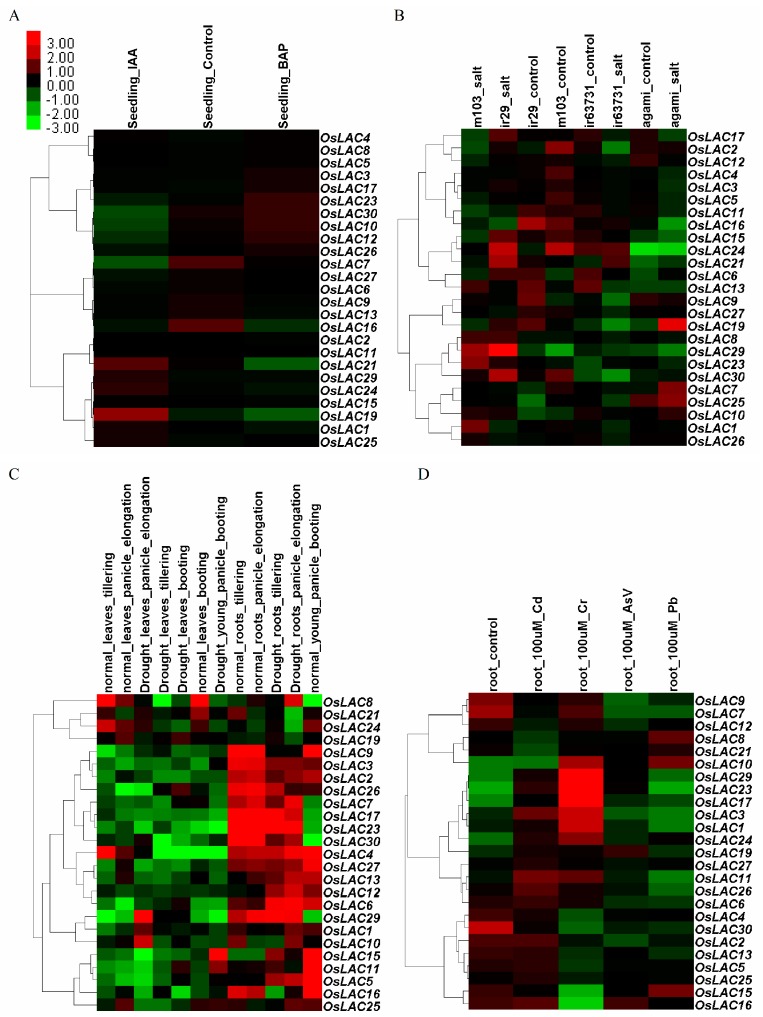
Clustering heat map of rice laccase genes expressions under hormones (**A**); salt (**B**); drought (**C**); and toxic heavy metals (**D**) treatments, Normalized Signal Intensity of genes obtained from Rice Oligonucleotide Array Database. Indica rice seedlings of ir64 variety were used for analysis of rice laccase genes expressions under hormones, drought and toxic heavy metals treatments; a salt-sensitive japonica rice m103, salt-tolerant japonica rice agami, salt-sensitive indica rice ir29 and salt-tolerant indica rice ir63731 were used for analysis of rice laccase genes expressions under salt treatment.

Abiotic stressors, including hormones, salt, drought, and toxic heavy metals (Cr, Cd, As, Pb) induced the expression of rice laccase genes. *OsLAC19* was significantly up-regulated under the treatment of indole-3-acetic acid (IAA) (Figure 4A). Two genes (*OsLAC7* and *OsLAC19*) were obviously induced by salt stress in tolerant japonica rice agami; *OsLAC21* was up-regulated in salt-treated tolerant indica rice ir63731. *OsLAC8*, *OsLAC10* and *OsLAC29* were significantly induced by salt stress in both salt-sensitive cultivars (japonica rice m103 and indica rice ir29) (Figure 4B). When rice suffered from drought stress, seven and four *OsLACs* were significantly up-regulated in the rice leaves and roots, respectively, at the tillering stage; *OsLAC6*, *OsLAC10* and *OsLAC21* were highly up-regulated both in the rice leaves and roots at the stage of panicle elongation (Figure 4C). Heavy metals can also influence the expression of *OsLACs*, three laccase genes (*OsLAC17*, *OsLAC23* and *OsLAC29*) were induced by Cr, Cd and As stresses; *OsLAC10* was up-regulated under Cr, As and Pb stresses, *OsLAC24* was up-regulated under Cr, Cd and Pb stresses, *OsLAC3* and *OsLAC11* were induced by Cr and Cd stresses, the expression level of *OsLAC1* and *OsLAC19* increased under Cr stress (Figure 4D). Taken together, some members of *OsLACs* family were up-regulated during various stresses. For example, *OsLAC10* can be induced by salt, drought and heavy metals stresses; *OsLAC19* was up-regulated under hormone, salt and heavy metals stresses.

### 2.4. Cu-Inducible Expression Patterns of Rice Laccase Genes

**Figure 5:**
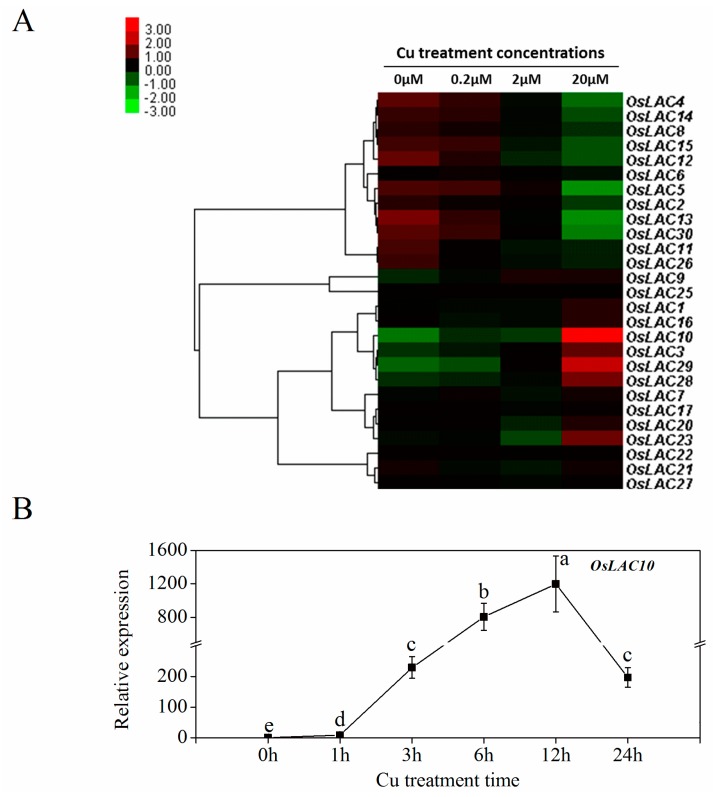
Expression of *OsLAC10* in rice roots under Cu stress. (**A**) Cluster heat map of rice laccase genes expressions under various Cu treatments; (**B**) qPCR analysis of the time course of *OsLAC10* expression in Cu (20 μM)-treated rice roots. The data are the means ± SD (*n* = 3). Data were ln(x + 1) transformed to achieve homogeneous variances before statistical analysis, different letters (a, b, c, d, e) above the column indicate a significant difference at *p* < 0.05 according to the least-significant difference (LSD) tests.

Because Cu stress was found to induce the activity of rice laccases [33], we performed a transcriptomic analysis of rice roots under Cu stress using RNA-Seq to examine the expression levels of the laccase genes under Cu stress. As shown in Figure 5A, the expressions levels of five rice laccase genes (*OsLAC3*, *OsLAC10*, *OsLAC23*, *OsLAC28*, and *OsLAC29*), particularly *OsLAC10*, were significantly induced by the 20 μM Cu treatment. The expression levels of another 11 *OsLAC*s decreased with increasing Cu concentration, and the other genes did not change significantly. A qRT-PCR analysis showed that the *OsLAC10* expression level increased significantly one hour after the 20 μM Cu treatment (Figure 5B) and peaked (about 1200-fold higher) at 12 h after the Cu treatment (compared to controls). These results indicate that *OsLAC10* is a typical Cu-induced gene in rice roots.

### 2.5. Expression of the Rice Laccase Protein OsLAC10 in Escherichia coli 

**Figure 6:**
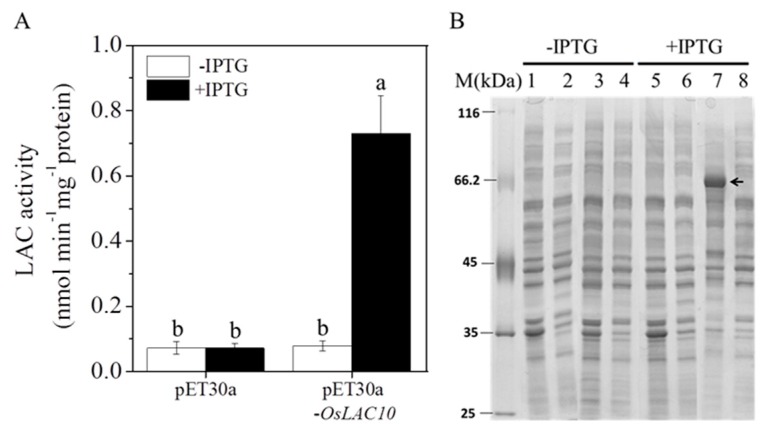
Expression of the OsLAC10 protein in *E*. *coli*. (**A**) Intracellular laccase activity of the *E*. *coli* BL21(DE3) strain expressing OsLAC10; (**B**) SDS-PAGE of the pET30a-*OsLAC10* recombinant protein; lanes 1 and 2, pET30a precipitate and supernatant; lanes 3 and 4, pET30a-*OsLAC10* precipitate and supernatant; lanes 5 and 6, pET30a precipitate and supernatant with 1 mM IPTG; lanes 7 and 8, pET30a-*OsLAC10* precipitate and supernatant with 1 mM IPTG. M, protein marker. Black arrows represent target proteins. The data are the means ± SD (*n* = 3). Different letters (a, b) above the column indicate a significant difference at *p* < 0.05 according to the LSD tests.

To detect the laccase activity of the *OsLAC10*-encoding protein, we constructed a recombinant pET30a-*OsLAC10* plasmid and transformed it into *E*. *coli* BL21 (DE3), with an empty plasmid (pET30a) as a control. After induction with isopropyl β-d-1-thiogalactopyranoside (IPTG), the laccase activity of the transgenic pET30a-*OsLAC10 E*. *coli* increased significantly to 10.2 times higher than that of the control. The laccase activities of *E*. *coli*-containing pET30a-*OsLAC10* and the empty plasmid were very low without IPTG induction (Figure 6A).

To further confirm the expression of the rice laccase protein OsLAC10 in *E*. *coli*, we used sodium dodecyl sulfate-polyacrylamide gel electrophoresis (SDS-PAGE) to detect the expression of the fusion protein. As shown in Figure 6B, after induction with IPTG, precipitation of transgenic pET30a-*OsLAC10 E*. *coli* resulted in a recombinant protein band (about 66 kDa). These results indicate that *OsLAC10* encoded a protein with laccase activity.

### 2.6. Ectopic Expression of OsLAC10 in Arabidopsis

**Figure 7:**
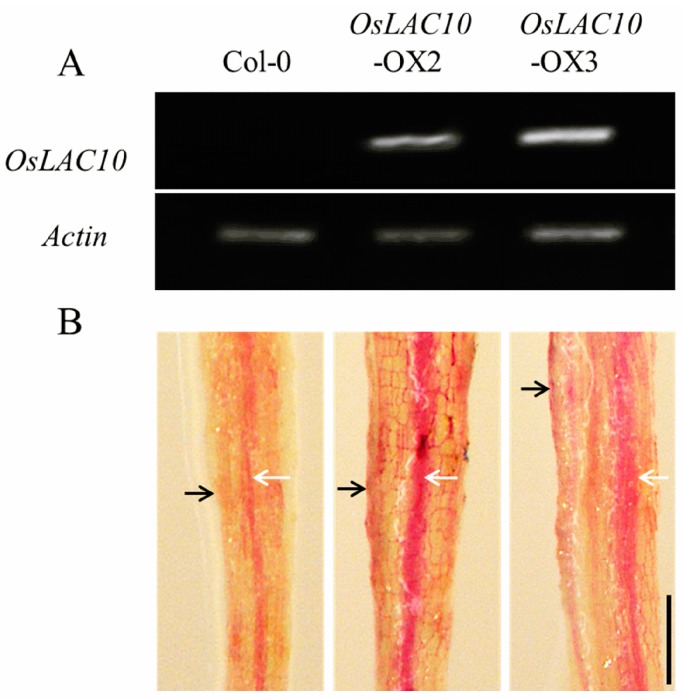
Transgene expression and lignin accumulation in transgenic *Arabidopsis* plants overexpressing *OsLAC10*. (**A**) Expression levels of *OsLAC10* in transgenic *Arabidopsis* plants; (**B**) Histochemical localization of lignin in root of wild-type and transgenic *Arabidopsis*. Lignified portion is red. Black and white arrows show epidermis and vascular cylinder, respectively. Black bars, 200 μm.

We overexpressed *OsLAC10* in *Arabidopsis* to investigate its biological function, and confirmed the presence of *OsLAC10* in *Arabidopsis* by RT-PCR (Figure 7A). Lignin levels in *OsLAC10* transgenic *Arabidopsis* roots were examined to determine the effects of overexpressing *OsLAC10* on lignin biosynthesis. We used lignin histochemical staining to determine lignin accumulation. As shown in Figure 7B, red staining detection of lignin was seen in epidermis and vascular cylinder in *Arabidopsis* roots. The red staining level of roots from the two *OsLAC10* transgenic *Arabidopsis* lines were stronger than that of the wild type, suggesting that *OsLAC10* is likely involved in lignin biosynthesis.

### 2.7. OsLAC10-Overexpressing Arabidopsis Shows Enhanced Cu Tolerance

**Figure 8:**
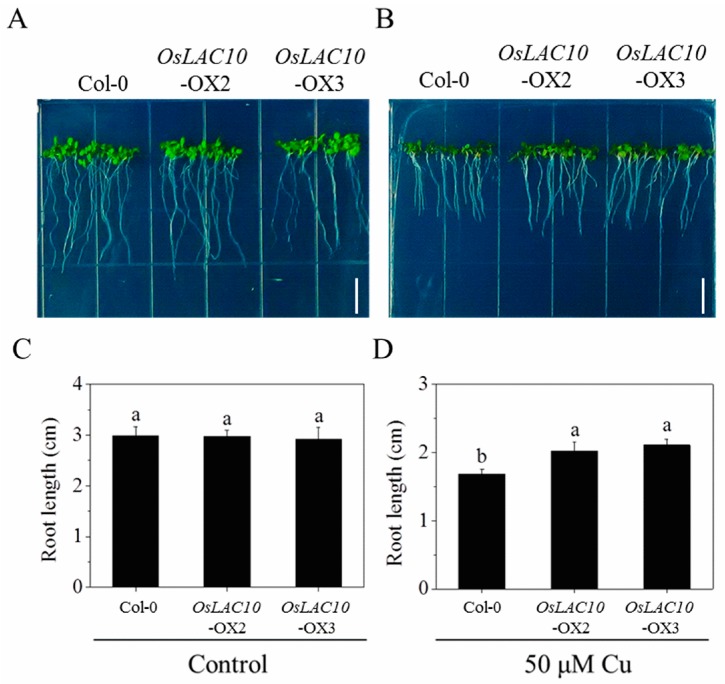
Effect of Cu stress on growth of wild-type and *OsLAC10* transgenic *Arabidopsis* on 1/2 Murashige and Skoog (MS) medium. (**A**,**B**) Growth phenotypes of wild-type and *OsLAC10* transgenic *Arabidopsis* under normal conditions and Cu (50 μM) stress, respectively, white bars, 1 cm; (**C**,**D**) Changes in root length of wild-type or *OsLAC10* transgenic *Arabidopsis* under control conditions and Cu (50 μM) stress, respectively. The data are the means ± SD (*n* = 8). Different letters (a, b) above the column indicate a significant difference at *p* < 0.05 according to the LSD tests.

To test whether overexpression of *OsLAC10* affects Cu tolerance, we grew wild-type and transgenic *Arabidopsis* on 1/2 Murashige and Skoog (MS) medium containing normal (0.1 μM Cu) or high levels of Cu (50 μM Cu). After growth on normal medium for seven days, the *OsLAC10* transgenic plants exhibited no differences in morphology or growth compared to the wild type (Figure 8A,C), whereas growth of *OsLAC10* transgenic *Arabidopsis* was greater than that of the wild type under 50 μM Cu treatment; the roots of *OsLAC10*-OX2 and *OsLAC10*-OX3 transgenic *Arabidopsis* were 20% and 25.2% longer than those of the wild type, respectively (Figure 8B,D).

### 2.8. Analysis of Cu Uptake and Translocation in Transgenic OsLAC10 Arabidopsis

**Figure 9:**
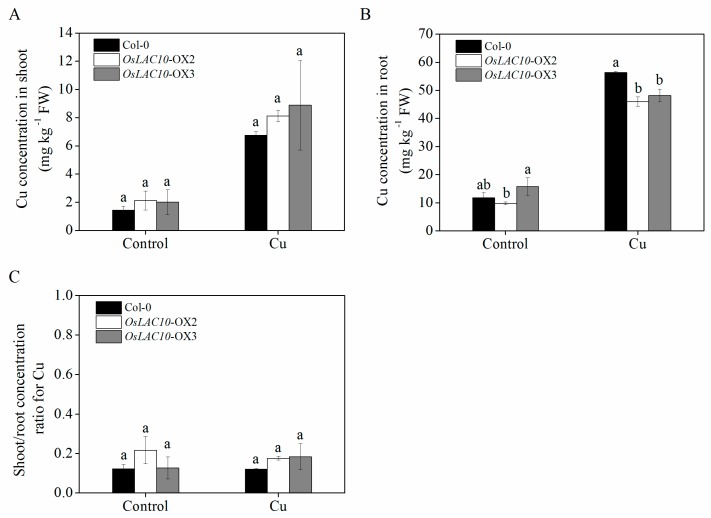
Cu concentration in shoots and roots of wild-type and *OsLAC10* transgenic *Arabidopsis* exposed to 5 μM Cu under hydroponic conditions. (**A**) Cu concentration in shoots; (**B**) Cu concentrations in root; (**C**) Cu transfer coefficient (S/R). The data are the means ± SD (*n* = 3). Different letters (a, b) above the column indicate a significant difference at *p* < 0.05 according to the LSD tests.

To further examine whether overexpression of *OsLAC10* in *Arabidopsis* affects uptake and translocation of Cu, we performed a hydroponic culture assay. Differences in copper accumulation were registered neither in control conditions nor after 5 μM Cu treatment, both in shoot of wild type and the *OsLAC10* transgenic lines (Figure 9A). In contrast, the *OsLAC10* transgenic *Arabidopsis* lines had significantly lower Cu concentrations in the roots than the wild type only under Cu (5 μM) stress conditions, but there was no significant difference in the Cu translocation coefficient between wild type and the transgenic lines (Figure 9B,C). These results suggest that overexpressing *OsLAC10* reduced the accumulation of Cu in *Arabidopsis* roots.

## 3. Discussion

To better understand of the function of rice laccase genes, we investigated their basic characteristics and evolutionary classification using bioinformatics. In agreement with previous reports on *Arabidopsis* laccases [17,34,35], we found that the rice laccase genes are mostly located in the secretory pathway, such as the cell wall and intercellular spaces. In addition, most have glycosylation sites for protein glycosylation, which ensures protein folding, stability, and formation of the cell wall (Table 1).

The rice laccase family can be divided into five subfamilies, similar to maize [36], whereas the *Arabidopsis* laccase family can be divided into six subgroups [17], indicating an evolutionary branching between monocots and dicots. The main function of laccase in plants is lignin biosynthesis; in *Arabidopsis*, such laccases include AtLAC4, AtLAC11, AtLAC15, and AtLAC17 [14,15,19]. AtLAC4 and AtLAC11 belong to group 2, AtLAC15 and AtLAC17 are in groups 4 and 1, respectively, indicating that they may be involved in lignin biosynthesis.

In our study, different laccase genes had various expression patterns in rice. The rice laccase genes were mostly expressed in roots at the vegetative growth stage, where they may be involved in root elongation and thickening. The expression patterns of laccase genes have been reported for a variety of other plants, particularly *A. thaliana* [8,11,17,36,37]. Many *Arabidopsis* laccase genes are expressed in roots and vascular tissues [38]. Although the expression patterns of these genes can be obtained by RT-PCR and microarray data analysis, their physiological functions are still largely unknown. We used RNA-seq and microarray analyses to investigate the expression patterns of rice laccase genes under hormone, salt, drought and heavy metal stressors. Some laccases are induced by a unique stress, whereas others, such as *OsLAC10*, respond to a variety of stressors. In *Arabidopsis*, *LAC1* can be induced by drought, oxidation, and high temperatures, whereas cold injury, osmotic, and salt stress induce the expression of *LAC14* [17].

Cu is a heavy metal and a laccase cofactor that is very important for its catalytic function. Expression of some laccase genes is closely related to the Cu level in plants [17]. The expression of miRNA of some laccase genes in *Arabidopsis* is downregulated under Cu-deficient conditions [39]. Our transcriptomic analysis showed that Cu stress strongly induced the expression of laccase genes in rice roots, particularly for *OsLAC10*. Like most plant laccases, OsLAC10 is predicted to have an N-terminal cleavable signal peptide targeting it to the secretory pathway, after being secreted into the apoplast, OsLAC10 is likely to participate in extracellular lignin formation, which is involved in Cu detoxification in rice roots [33], indicating that *OsLAC10* may play an important role in response to Cu stress of rice roots.

Because many laccase proteins are putative in rice, we expressed *OsLAC10* in *E*. *coli*, and showed for the first time for rice laccases that the protein encoded by *OsLAC10* had the laccase activity. Sterjiades et al. purified the laccase protein ApLAC1 from a suspension of Eurasian maple cells and reported oxidization of lignin monomers from a water-insoluble polymer in vitro [5]. Cotton laccase gene *GaLAC1* encodes a secreted protein with laccase activity and participates in the degradation of trichlorophenol [10]. *TT10* encodes a laccase protein in the *Arabidopsis* seed coat that oxidizes flavonoids [40]. The *Rhus* laccase RvLAC2 oxidizes catechols, which participate in wound repair [41]. A Norway spruce laccase has been purified and biochemically characterized from a lignin-forming cell culture and has an acidic pH that is optimum for coniferyl alcohol oxidation [42]. These studies suggest that plant laccases catalyze a variety of substrate oxidation reactions in many physiological processes.

We collected some *OsLAC10* mutants to study the biological functions of *OsLAC10* in rice; however, no differences were detected between the wild type and mutants (data not shown), which may be due to the functional redundancy of rice laccases. The same was found in *Arabidopsis* [17]. Thus, we overexpressed *OsLAC10* in Arabidopsis, and found the more lignin accumulation in transgenic Arabidopsis roots compared with the wild-type by lignin staining. Therefore, we hypothesize that the *OsLAC10* gene likely promotes lignin biosynthesis. In *Arabidopsis*, *lac4-2 lac17* mutant had lower lignin content than wild-type, and lignin was not detected in the roots of the *Arabidopsis* laccase triple mutant (*lac4-2 lac11-1 lac17-1*), whereas wild-type roots showed normal lignin accumulation, indicating that *AtLAC4*, *AtLAC11* and *AtLAC17* are functional lignin laccase genes [14,15].

The role of *OsLAC10* in Cu tolerance as a Cu-induced gene in *Arabidopsis* was analyzed. The phenotypes of the transgenic lines were the same as the wild type in control medium, which may be due to there being no large changes in lignin accumulation in the transgenic lines compared with the wild type [14,43,44]. However, the transgenic lines grew better than the wild type on medium containing Cu, particularly the roots, suggesting that overexpression of *OsLAC10* enhanced Cu tolerance in *Arabidopsis*. In addition, absorption of Cu decreased in the transgenic lines, compared to the wild type, possibly because more lignin in transgenic *Arabidopsis* roots prevented Cu from entering the root cells. Few studies have investigated the roles of laccases in the responses of plants to stress. In a previous study, mutations in three laccase genes inhibited *Arabidopsis* root growth during PEG-induced drought stress [18]. Cho et al. reported that overexpression of a rice laccase gene *OsChI1* (Os01g61160) enhances salt and drought tolerance in *Arabidopsis*, which may be associated with the production of some phenolic polymers, and enhances flavonoid oxidation [20].

In conclusion, *OsLAC10* is a laccase gene that enhances the tolerance of *Arabidopsis* to Cu stress, possibly through lignification in roots that prevents excess absorption of Cu. In future research, functional analyses of rice multiple laccase genes mutants may provide more information about the biological functions of the rice laccase genes.

## 4. Materials and Methods

### 4.1. Plant Material and Treatments

Seeds of rice (*Oryza sativa* spp*. japonica* cv. Nipponbare) were sown on mesh floating on a 0.5 mM CaCl_2_ solution for 2 days at 30 °C in darkness, thereby inducing germination. Seedlings were transferred to Kimura B nutrient solution held under normal greenhouse conditions under illumination provided by cool-white fluorescent lamps [30]. The growth conditions were as follows: 27/24 °C day/night temperatures, 60%–80% relative humidity, and 14/10-h day/night photoperiod. 15-day-old rice seedlings (shoot and root) and 3-month-old rice flowers (stamen, pistil, palea and lemma) were used for quantitative RT-PCR analysis of expression patterns of rice *LAC* genes in various tissues/organs and developmental stages. For quantitative RT-PCR analysis of Cu-inducible expression patterns of rice laccase genes, 15-day-old rice seedlings were treated with 20 μM CuSO_4_ for 0, 1, 3, 6, 12 and 24 h, root samples from each treatment were harvested, immediately frozen and stored at −80 °C.

*Arabidopsis thaliana* ecotype Columbia-0 was used as the wild-type plant in this study. For Cu tolerance experiment, *Arabidopsis* seeds were surface-sterilized and stratified for 3 days in the dark at 4 °C. Then, *Arabidopsis* seeds were sown on 1/2 Murashige and Skoog (MS) medium [45] containing 50 μM CuSO_4_ or not for the Cu tolerance experiment, and root lengths were determined 1 week later. In the Cu accumulation experiment, *Arabidopsis* seeds were sown on 1/2-MS solid medium for germination. After 2 weeks, the seedlings were transferred to 1/4-Hoagland nutrient solution for 3 weeks and treated with CuSO_4_ (5 μM) for 3 days. A normal nutrient solution was used as the control. The sown seeds and the *Arabidopsis* seedlings were grown in an incubator with cool white fluorescent lights (Ningbo Saifu Experimental Instrument Co., Ltd., Ningbo, China), under a 16 h light/8 h dark photoperiod at 22/18 °C (day/night).

### 4.2. Identification of the Rice Laccase Genes (OsLACs)

The Arabidopsis laccase (AtLACs) amino acid sequences were obtained from the Arabidopsis information resource (TAIR, http://www.arabidopsis.org/index.jsp). We BLAST searched the National Center of Biotechnology Information (NCBI, http://www.ncbi.nlm.nih.gov/guide/), Universal Protein (Uniprot, http://www.uniprot.org/), Rice Genome Annotation Project (RGAP, http://rice.plantbiology.msu.edu/), and the Rice Annotation Project Database (RAP-DB, http://rapdb.dna.affrc.go.jp/) databases, using the conserved *Arabidopsis* laccase amino acid sequence, to find information on rice laccases, including amino acid sequences, gene loci, and chromosomal locations. MapInspect software (Ralph van Berloo, Wageningen, Netherlands) was used to generate a distribution map of *OsLACs* based on the location information of each laccase gene on rice chromosome.

### 4.3. Sequence Analysis and Phylogenetic Tree

The putative signal sequences, subcellular localization, and potential glycosylation sites were analyzed using SignalP, TargetP, and NetNGlyc 1.0 online program (http://www.cbs.dtu.dk/), respectively. Sequence and alignment analyses of rice laccases were performed with ClustalW (http://www.genome.jp/tools/clustalw). The sequences of poorly aligned positions and divergent regions were eliminated by using Gblocks Server (http://molevol.cmima.csic.es/castresana/Gblocks_server.html). The sequence identity matrix was calculated using BioEdit 7.1.3 software (Thomas A Hall, Raleigh, NC, USA). A phylogenetic tree was constructed using PHYML online execution program (http://atgc.lirmm.fr/phyml/), according to the method of Caparrós-Ruiz et al. [36]. Mega 6.0 software (Sudhir Kumar, Phoenix, AZ, USA) was used to edit the tree.

### 4.4. Expression Analysis of Rice Laccase Family Genes

The normalized spatiotemporal expression signal intensities of the rice laccase genes (Table S2) were obtained from the Rice Expression Profile Database (RiceXPro, http//ricexpro.dna affrc.go.jp/category-select.php). The normalized signal intensities of rice laccase gene expressions under various stressors (hormones, drought, salt and toxic heavy metals) (Tables S3–S6) were obtained from the Rice Oligonucleotide Array Database (http://www ricearray.org/), including four experiments: GSE5167 (Rice seedling hormone treatment), GSE4438 (Expression data from rice under salinity stress), GSE26280 (Genome-wide temporal-spatial gene expression profiling of drought), and GSE25206 (Transcriptomic shifts in rice roots in response to Cr (VI) stress). The experiment GSE5167 involved the identification of differentially expressed genes in IAA and BAP treated indica rice seedlings of ir64 variety as compared to control [46]; in the experiment GSE4438, a salt-sensitive japonica rice m103, salt-tolerant japonica rice agami, salt-sensitive indica rice ir29 and salt-tolerant indica rice ir63731 were used for expression analysis using the tissue from crown and growing point under control and salt stressed conditions at the sensitive early reproductive stage (panicle initiation) [47]; in the experiment GSE26280, the gene expression patterns across six tissues including leaves and roots at tillering stage and panicle elongation stage, leaves and young panicle at booting stage were characterized by using the Affymetrix rice microarray platform based on a drought tolerant rice line derived from ir64 [48]; in the experiment GSE25206, indica rice ir64 seedlings treated with 100 μM of Cr (VI), As (V), Cd, and Pb were used for analysis of genome-wide transcriptome profiling in rice root in response to heavy metal stress [49]. *OsLAC* expression in rice roots under Cu stress were obtained from our previous expression data (Table S7), which from RNA-Seq analysis of 15-day-old rice seedlings roots under different Cu treatment (0, 0.2, 2 and 20 μM) for 24 h, the detailed data analysis method can be found in our recent published paper [50]. Cluster 3.0 software (Michiel de Hoon, Tokyo, Japan) was used to build a cluster heat map of laccase gene expression.

### 4.5. Quantitative RT-PCR Analysis

Total RNA for qRT-PCR analyses was extracted using a plant RNA extraction kit (TaKaRa Bio, Dalian, China). RNAs were reverse-transcribed using PrimeScript RT Master Mix (TaKaRa Bio, Dalian, China), cDNAs were amplified with SYBR pre-mix EX Taq (TaKaRa Bio, Dalian, China), and the qRT-PCR was performed on a 7500 PCR system (Applied Biosystems, Waltham, MA, USA) with the primer for *OsLAC10* shown in Table S8. The PCR protocol was as follows: initial denaturation at 95 °C for 30 s, followed by 95 °C for 5 s, and 60 °C for 34 s in a 40-cycle reaction.

### 4.6. Plasmid Construction and Generation of Transgenic E. coli and Arabidopsis

To obtain the recombinant OsLAC10 protein expressed in *E*. *coli*, pET30a-*OsLAC10* was generated by cloning the full-length coding sequence (CDS, 1740 bp) of the *OsLAC10* gene into the pET30a vector at the *Eco*R V and *Eco*R I cloning sites. The *OsLAC10* CDS was cloned into the pBI121 plant binary expression vector at the Xba I/Sma I cloning sites and transformed into wild-type *Arabidopsis* (Columbia-0) via the floral dip method using *Agrobacterium tumefaciens* (EHA105) [51]. The transgenic plants were screened according to the method of Lv et al. [52]; homozygotic transgenic lines of T3 progeny were used for *OsLAC10* function study. For transgene expression analysis, semi-quantitative RT-PCR was performed, the PCR protocol was as follows: denaturing for 3 min at 94 °C, followed by 30 cycles of 30 s of denaturation at 94 °C, 30 s of annealing at 58 °C, and 60 s of extension at 72 °C, with a final extension step at 72 °C. The primers used for plasmid construction and generation of transgenic *E*. *coil* and *Arabidopsis* are shown in Table S8.

### 4.7. Transgenic E. coli Laccase Activity Measurement and SDS-PAGE of Recombinant Proteins

Frozen BL21 (DE3) containing the recombinant plasmid and empty plasmid was inoculated on solid yeast extract broth (YEB) medium, and picked monoclonal inoculated in liquid Luria-Bertani broth (LB) medium (containing 50 μg/mL Kanamycin) at 37 °C with shaking overnight. Then expanding culture by a ratio of 1:100 in the above-mentioned medium at 37 °C with 220 rpm shaking until OD_600_ reached to 0.6–0.8. IPTG was added to a final concentration of 1 mM, shaking for 5 h at 37 °C, then centrifuged bacterial fluid at 6000 rpm for 15 min at 4 °C. Thallus were collected, resuspended in 20 mM Tris-HCl (pH 8.0) and broken by the ultrasonic wave. After centrifuging at 12,000 rpm for 15 min, the precipitated fraction was suspended with 20 mM Tris-HCl (pH 8.0). Laccase activity was determined in 30 μL supernatant by monitoring the oxidation of 2,2′-Azinobis-(3-ethylbenzthiazoline-6-sulphonate) (ABTS) (Sigma-Aldrich, St. Louis, MO, USA) at 420 nm, according to the method of Wang et al. [53].

After protein quantification, the suspension and supernatant was mixed the SDS-PAGE loading buffer, respectively, heated at 100 °C for 3 min, 20 μL protein solution was loaded, after electrophoresis, the gel was stained by silver nitrate and photographed.

### 4.8. Lignin Staining and Determination of Cu Concentrations of Transgenic Arabidopsis

The method of Lequeux et al. [54] was used to stain lignin in 3-week-old *Arabidopsis* roots, which were stained with 1% (*w*/*v*) phloroglucinol in 6 N HCl for 5 min, then were photographed by a stereo microscope (Nikon SMZ1000, Tokyo, Japan).

Cu^2+^ concentration was determined according to the method of [33]. In brief, the plant material (roots and shoots) were digested in heat-resistant glass tubes in a heating block with a mixture of HNO_3_-HClO_4_ (87:13 *v*/*v*). The digests were eventually dissolved in 5% HNO_3_ for metal analyses using ICP-OES (Optima 2100DV, PerkinElmer, Waltham, MA, USA).

### 4.9. Statistical Analysis

The data were analyzed by one-way analysis of variance (ANOVA), followed by mean comparisons with LSD tests at the *p* < 0.05 significance level, using SPSS (Statistical Package for Social Science for Windows, ver. 13.0, Somers, New York, NY, USA) software. Data were ln(x + 1) transformed before statistical analysis when variances were not homogeneous, although the non-transformed data are presented.

## Figures and Tables

**Table 1:** Gene locus and prediction of N-terminal signal peptides and glycosylation sites of rice laccases.

Gene Name	Locus Number	Amino Acids Length	Predicted Target Site	Signal Peptide Length	Cleavage Site	Number of *N*-glycosyl Sites
*OsLAC1*	Os01g0374600	599	Secretory	28	AHG-AK	7
*OsLAC2*	Os01g0634500	562	Secretory	26	AHA-DV	13
*OsLAC3*	Os01g0827300	567	Secretory	24	AGA-EV	8
*OsLAC4*	Os01g0842400	579	Secretory	28	AQG-IT	14
*OsLAC5*	Os01g0842500	577	Secretory	27	AKG-DI	11
*OsLAC6*	Os01g0843800	547	Mitochondrion	35	TAG-LT	10
*OsLAC7*	Os01g0850550	580	Secretory	22	AQA-DV	6
*OsLAC8*	Os01g0850700	559	Secretory	28	ADA-AT	15
*OsLAC9*	Os01g0850800	554	Secretory	21	ASA-AV	5
*OsLAC10*	Os02g0749700	579	Secretory	21	ALA-VN	6
*OsLAC1* *1*	Os03g0273200	578	Secretory	29	AGA-AT	15
*OsLAC12*	Os03g0297900	646	Secretory	33	AVA-EE	3
*OsLAC13*	Os05g0458300	513	-	-	-	-
*OsLAC14*	Os05g0458500	549	Secretory	27	AEA-IT	11
*OsLAC15*	Os05g0458600	574	Secretory	27	AEA-IT	12
*OsLAC16*	Os07g0101000	583	Secretory	30	VDA-AI	11
*OsLAC17*	Os10g0346300	599	Secretory	-	-	-
*OsLAC18*	LOC_Os10g30120	69	Secretory	24	TNY-TR	2
*OsLAC19*	Os10g0437400	467	Mitochondrion	28	VDQ-PR	5
*OsLAC20*	Os11g0108650	201	Secretory	22	AAA-KE	1
*OsLAC21*	Os11g0108700	326	-	-	-	8
*OsLAC22*	Os11g0264000	595	Secretory	29	GEA-AV	8
*OsLAC23*	Os11g0641500	590	Secretory	28	GEA-AV	8
*OsLAC24*	Os11g0641800	580	Secretory	23	GEA-GV	8
*OsLAC25*	Os11g0696900	583	Secretory	29	AHG-GR	8
*OsLAC26*	Os11g0708100	586	Chloroplast	-	-	-
*OsLAC27*	Os12g0108000	567	Secretory	22	AAA-KE	11
*OsLAC28*	Os12g0257600	571	Secretory	18	AHG-AV	13
*OsLAC29*	Os12g0258700	579	Secretory	23	AQA-AV	14
*OsLAC30*	Os12g0259800	577	Secretory	22	AQA-AV	10

“-” represent any other location or not detected.

## Supplementary Materials

Supplementary materials can be found at www.mdpi.com/1422-0067/18/1/209/s1.
